# Healthcare Goes Digital: mHealth, eHealth, Artificial Intelligence, and Emerging Digital Technologies Within Digital Health Transformation

**DOI:** 10.3390/healthcare14091173

**Published:** 2026-04-28

**Authors:** Daniele Giansanti

**Affiliations:** Centro Nazionale IATIS, Istituto Superiore di Sanità, Via Regina Elena 299, 00161 Roma, Italy; daniele.giansanti@iss.it; Tel.: +39-06-49902701

Following the success of the first Special Issue, “*The 10th Anniversary of Healthcare—TeleHealth and Digital Healthcare*” [1], the journal continues its focus on the structural transformation of healthcare systems driven by digital innovation with the present second edition, “*Healthcare Goes Digital: Mobile Health and Electronic Health Technology in the 21st Century: Second Edition*” [2]. The strong scientific response to the first edition confirmed that digital health is no longer experimental but an integral component of contemporary healthcare delivery.

Mobile health (mHealth), electronic health (eHealth), and telemedicine have become established elements of healthcare systems, enabling remote care delivery, continuous monitoring, and improved patient engagement. Recent systematic evidence suggests that digital health interventions can improve clinical outcomes and healthcare utilisation, particularly when integrated into routine care pathways and supported by appropriate implementation strategies [3]. Telemedicine has demonstrated effectiveness across clinical domains, particularly in chronic disease management and outpatient care, supporting its role in improving access and continuity of care [4].

Artificial intelligence (AI) is increasingly shaping the evolution of digital healthcare systems [5,6,7,8]. Evidence from recent studies highlights its expanding role in diagnostic support, risk prediction, and clinical decision-making, while also emphasising key limitations related to bias, generalisability, and regulatory readiness [5,6,7,8]. In parallel, wearable devices and remote monitoring technologies are enabling continuous physiological data collection, supporting a shift toward preventive and personalised medicine.

More recently, generative AI and large language models (LLMs) have introduced new capabilities in healthcare, including clinical documentation support, information summarisation, and patient interaction [9]. Early peer-reviewed evidence suggests that these systems can achieve high performance in medical knowledge tasks, while also raising concerns regarding factual reliability, hallucination risk, and safe integration into clinical workflows [10].

This Special Issue [2] aimed to foster a rigorous interdisciplinary discussion on the convergence of mHealth, eHealth, AI, and emerging digital technologies, embracing mobile health applications, wearable systems, telemedicine, electronic health records, data governance, cybersecurity, patient engagement, and the responsible integration of generative AI in healthcare.

The Special Issue includes ten works, comprising nine papers (Contributions 1–9), which consist of seven original research articles (Contributions 1–7), one protocol (Contribution 8), and one systematic review (Contribution 9), together with this concluding editorial.

To better frame the scope and implications of the contributions included in this Special Issue, a (1) cross-cutting synthesis is presented first, followed by (2) a detailed overview of each individual contribution, and (3) a final synthesis highlighting the main implications of the Special Issue and outlining future directions for research and the responsible advancement of digital healthcare systems.

*Cross-cutting synthesis of the Special Issue*

Taken together, the nine contributions illustrate a coherent yet heterogeneous transformation of digital healthcare, in which emerging technologies such as artificial intelligence, mHealth, telemedicine, and digital clinical support systems are increasingly embedded within care delivery and professional practice rather than functioning as isolated tools.

A first cross-cutting theme concerns the acceptance, adoption, and implementation barriers of digital health technologies, including AI-supported medical history-taking (Contribution 1), digital cytology workflows (Contribution 7), and telehealth literacy determinants (Contribution 6). These studies consistently highlight the central role of human, organisational, and cultural factors in shaping the integration of digital solutions into clinical practice, while also suggesting the presence of gaps between technological potential and effective adoption, particularly in relation to user trust, digital competencies, and system-level readiness.

A second theme relates to the reconfiguration of care and education models, where digitally mediated approaches such as Project ECHO (Contribution 2), mHealth interventions (Contribution 5), and telematic rehabilitation protocols (Contribution 8) demonstrate the potential to expand access to care, improve continuity, and support more distributed and patient-centred models of healthcare delivery. At the same time, these contributions reflect variability in study design and level of evidence, ranging from pilot studies and protocols to more structured implementations, highlighting the need for further validation and long-term outcome assessment.

A third dimension concerns the integration of artificial intelligence into clinical and decision-support processes, as illustrated by automated cognitive and mood assessment systems (Contribution 4), AI-supported diagnostic and workflow applications (Contributions 1 and 7), and computer-assisted navigation technologies in surgery (Contribution 9). While these contributions collectively underline the transformative potential of AI, they also point to ongoing challenges related to validation, generalisability across settings, and the safe and effective translation of AI-based tools into routine clinical practice.

Finally, the contributions converge towards a broader paradigm of data-driven, preventive, and personalised digital healthcare, where continuous monitoring, digital assessments, and scalable frameworks (Contribution 3) enable more proactive and longitudinal approaches to patient management. At the same time, they indicate areas for further investigation, particularly in relation to integration across contexts, scalability, and long-term implementation within real-world healthcare systems.

*Detailed synthesis of the Special Issue contributions*

The nine contributions collectively explore the ongoing transformation of healthcare systems through digital technologies, including artificial intelligence, telemedicine, mHealth, and digital clinical workflows.

Contribution 1 examines the acceptance of AI-supported medical history-taking and chatbot-based anamnesis in the German population, focusing on attitudes towards digital tools in clinical history collection and patient interaction. Contribution 2 explores Project ECHO as a digitally mediated telementoring model designed to expand access to specialist expertise and support continuing professional development through structured remote learning environments.

Contribution 3 presents a feasibility and proof-of-concept study on the digitalisation of Comprehensive Geriatric Assessment (CGA) in nursing practice, with potential application in long-term care settings and nursing homes. Contribution 4 introduces an automated system for cognitive and mood assessment in hyper-acute stroke units, highlighting the role of digital tools in standardised clinical evaluation in acute neurological care.

Contribution 5 reports a pilot study evaluating an mHealth-based occupational therapy intervention aimed at improving functional performance in individuals with neurodevelopmental disorders. Contribution 6 analyses telehealth literacy among users and examines demographic and behavioural factors associated with differences in digital health competencies and access to telehealth services.

Contribution 7 examines the integration of AI into digital cytology workflows in Italy, focusing on professional perceptions and implementation-related aspects. Contribution 8 presents a study protocol for a randomised controlled trial evaluating home-based telematic exercise interventions for patients with permanent colostomy, addressing postoperative rehabilitation through digital approaches. Finally, Contribution 9 provides a systematic review of dynamic computer-aided navigation systems in dentoalveolar surgery and maxillary bone augmentation, focusing on clinical application and methodological characteristics.

*Closing synthesis and lessons for the future*

These nine contributions collectively outline a coherent and multidimensional transformation of digital healthcare, spanning clinical practice, education, rehabilitation, and diagnostic support systems. They highlight the progressive integration of digital technologies such as artificial intelligence, mHealth, telemedicine, and digital clinical workflows into healthcare delivery and organisation, moving from experimental or isolated applications towards more structured and embedded components of care pathways.

At the same time, the evidence emerging from this Special Issue suggests that technological advancement alone may not be sufficient to ensure effective implementation. Human, organisational, and cultural factors, together with aspects related to governance, usability, and professional acceptance, appear to play a central role in shaping adoption and potential impact across different healthcare contexts.

From this perspective, several considerations for future developments emerge. First, the integration of digital health solutions may benefit from closer alignment between technological design and real-world clinical workflows. Second, attention to digital literacy, professional training, and end-user engagement appears relevant to support the more effective and inclusive use of these tools. Third, the expansion of artificial intelligence and data-driven systems highlights the importance of ongoing evaluation in terms of reliability, generalisability, and safety, particularly in high-stakes clinical environments. Finally, the contributions point towards the potential value of interdisciplinary collaboration in supporting the responsible and patient-centred evolution of digital healthcare systems.
